# Core Concepts in Pharmacoepidemiology: New‐User Designs

**DOI:** 10.1002/pds.70048

**Published:** 2024-11-25

**Authors:** Qoua L. Her, Julie Rouette, Jessica C. Young, Michael Webster‐Clark, John Tazare

**Affiliations:** ^1^ Department of Epidemiology, Gillings School of Global Public Health University of North Carolina at Chapel Hill Chapel Hill North Carolina USA; ^2^ GSK Montreal Quebec Canada; ^3^ Unit of Epidemiology, Institute of Environmental Medicine Karolinska Institutet Stockholm Sweden; ^4^ Department of Epidemiology, Biostatistics, and Occupational Health McGill University Montreal Quebec Canada; ^5^ Department of Medical Statistics London School of Hygiene & Tropical Medicine London UK

**Keywords:** active comparator new user design, new‐user design, pharmacoepidemiology, prevalent new user design

## Abstract

In this article, we review the history and key reasons for new‐user comparisons in pharmacoepidemiology, highlighting the target trial framework as a unifying framework. We describe three distinct pharmacoepidemiological new‐user study designs: (1) new‐user versus non‐user; (2) active comparator new‐user; (i.e., ACNU) and (3) prevalent new‐user (i.e., PNU) designs, and discuss how each relates to key issues of defining time zero, choosing appropriate comparator groups, and potential sources of bias they do and do not account for. We use a clinical example of hormone replacement therapy and the risk of coronary heart disease to illustrate practical considerations surrounding the application of the three designs presented.

**Summary**

- The article describes the use of new‐user versus non‐user, active comparator new‐user (i.e., ACNU) and prevalent new‐user (i.e., PNU) designs in pharmacoepidemiology while emphasizing how these designs align with the target trial framework.
- We describe causal questions that can be studied using these designs through the lens of a hormone replacement therapy example.
- The new‐user versus non‐user design focuses on causal questions related to mimicking initiation of treatment versus placebo, ensuring clear temporality between covariates and treatment initiation whilst avoiding bias due to the inclusion of prevalent users.
- The active comparator new‐user design compares patients initiating treatment against those initiating a **clinically relevant** alternative to minimize **confounding by indication**.
- The prevalent new‐user design expands the population of inference to include patients who switch treatment by incorporating comparisons to those who continue treatment to mitigate issues arising from the inclusion of prevalent users.

## 1 Introduction

As pharmacoepidemiologists, we evaluate medical interventions in populations and provide evidence to inform treatment and regulatory decisions. Observational study designs leveraging real‐world data generated from interactions with the healthcare system (e.g., from administrative claims and electronic medical records) are often the most efficient and suitable way to inform those decisions by answering causal questions related to medical interventions.

Some of the most frequently asked of these causal questions include whether to start a treatment, which treatment to choose between therapeutic options, and whether to switch from a current treatment to a newly available therapeutic alternative. These types of causal questions correspond to three common pharmacoepidemiologic new‐user study designs: the “new‐user versus non‐user design,” [1, 2] the “active comparator new‐user (i.e., “ACNU”) design,” [1, 2, 3, 4] and the “prevalent new‐user (i.e., “PNU”) design,” respectively [5]. First, the “new‐user versus non‐user design,” examines whether patients should initiate treatment. It emphasizes the importance of key underlying principles, relevant to all new‐user designs: following all patients in a study from the time of treatment initiation, determining a clear timeline for measurement of baseline covariates prior to initiation, enabling the study of early effects of treatment, and avoiding biases related to poor persistence. The “active‐comparator new‐user design,” originally described using different terminology in a seminal paper on incident user designs [2], was later used to delineate a set of questions addressing which of two (or more) active treatment options is safer or more effective. The active‐comparator new‐user study design can be more clinically relevant when there are two approximately equivalent treatment options available and helps address baseline confounding by comparing patients with the same (or very similar) indications for treatment [6]. Most recently, the “prevalent new user design” was introduced to address a different clinical question of whether a patient who is already on one type of treatment should switch or augment existing treatment with a new treatment option [5]. This final study design emphasizes populations that are not typically included in new‐user based study designs.

The “target trial framework” is growing in popularity and frames observational research on safety and effectiveness as an attempt to mimic a randomized trial. [7, 8, 9] This framework can be used to generate evidence for decision‐making in many of the same situations where a randomized trial would be applied, provided that the data are detailed enough to emulate the trial. Importantly, each of the aforementioned study designs corresponds to distinct target trials answering distinct causal questions.

The aim of this article is to describe use cases for the new‐user versus non‐user [1, 2], active comparator new‐user [3, 4], and prevalent new‐user designs [5] in the context of the target trial framework and a simple illustrative example. By understanding the strengths of the designs and the target trials underlying them, researchers can choose the most appropriate study design to answer their specific causal question.

## 2 Illustrative Example

The cardiovascular safety of hormone replacement therapy (HRT) is one of the most well‐known examples of discordant results between randomized controlled trials (RCTs) and observational studies. Despite multiple observational studies, including the Nurses' Health Study [10, 11, 12], suggesting a cardiovascular benefit to HRT use in post‐menopausal women, the Women's Health Initiative trial comparing HRT initiation to placebo found an increase in adverse cardiovascular events among HRT initiators [13]. One commonly cited reason for this discrepancy between the observational and experimental findings is that the “exposed versus unexposed person‐time” structure of the observational studies did not properly correspond to a hypothetical target trial and introduced a variety of potential biases [14]. Within the context of this example, we illustrate how each of the aforementioned three pharmacoepidemiologic study designs could be used to emulate distinct, well‐defined target trials. To better illustrate key elements of these study designs, we invoke a hypothetical novel HRT therapy, *Newstogen*, which was shown to have cardiovascular benefits in a placebo‐controlled Phase III RCT.

The key features of the presented study designs are summarized in Table 1, including information surrounding types of research questions that can be answered, comparator groups, and specification of time zero of follow‐up (i.e., index date or baseline).

**TABLE 1:** Features and considerations of presented study designs.

Design	Research question	Comparator groups	Defining time zero	Key considerations
New‐user versus non‐user design	Targets questions surrounding patients who, following a washout period, initiate treatment or do not initiate treatment (i.e., comparing users and non‐users of treatment). **HRT Example** Compare risk of CHD in initiators of Newstogen versus non‐users of HRT	Since we can only emulate pragmatic trials, the comparator group focuses on non‐users of treatment (as opposed to those receiving a placebo). Can also be described as ‘untreated’	For the treated group, time zero is the time of treatment initiation For the untreated group, time zero is usually either: i. the first time a untreated patient is eligible ii. matched or randomly sampled based on the treatment initiation times of the treated group	Confounding by indication can be difficult to address. Incorporating non‐users can be difficult and, when done inappropriately, can introduce immortal time bias Restriction to new users reduces sample size and the precision of estimated treatments effects (compared with approaches including person‐time of individuals with prior use) Limited ability to study treatment switching or augmentation
Active comparator new‐user design	Targets questions surrounding patients who, following a washout period, initiate treatment A or initiate a clinically equipoise treatment **HRT Example** Compare risk of CHD in initiators of *Newstogen* versus initiators of an existing alternative HRT formulation	Clinically equivalent treatment	Time of treatment initiation	Requires the existence of a suitable active comparator Sample size limitations of new‐user designs can be exacerbated since new use is required for both treatment and comparator groups. Limited ability to study treatment switching or augmentation.
Prevalent new‐user design	Targets questions surrounding the treatment effect in those moving from a comparator treatment to a treatment of interest. **HRT Example** Compare risk of CHD in those switching to or initiating Newstogen versus those continuing on or initiating other HRT	Initiation of (or continuation on) clinically equivalent treatment	For the treatment of interest: time of initiation For new users of the comparator treatment: time of initiation For “continuation” observations of the comparator treatment: time of therapy continuation	Requires complex analytic methods which can introduce left truncation bias and depletion of susceptibles if not appropriately applied. Necessary to clarify the types of switchers (direct vs. delayed vs. complex) under consideration and operationalize definitions in a way that effectively delineates these different types.

## 3 New‐User Versus Non‐User Design

### 3.1 Motivation

**FIGURE 1:**
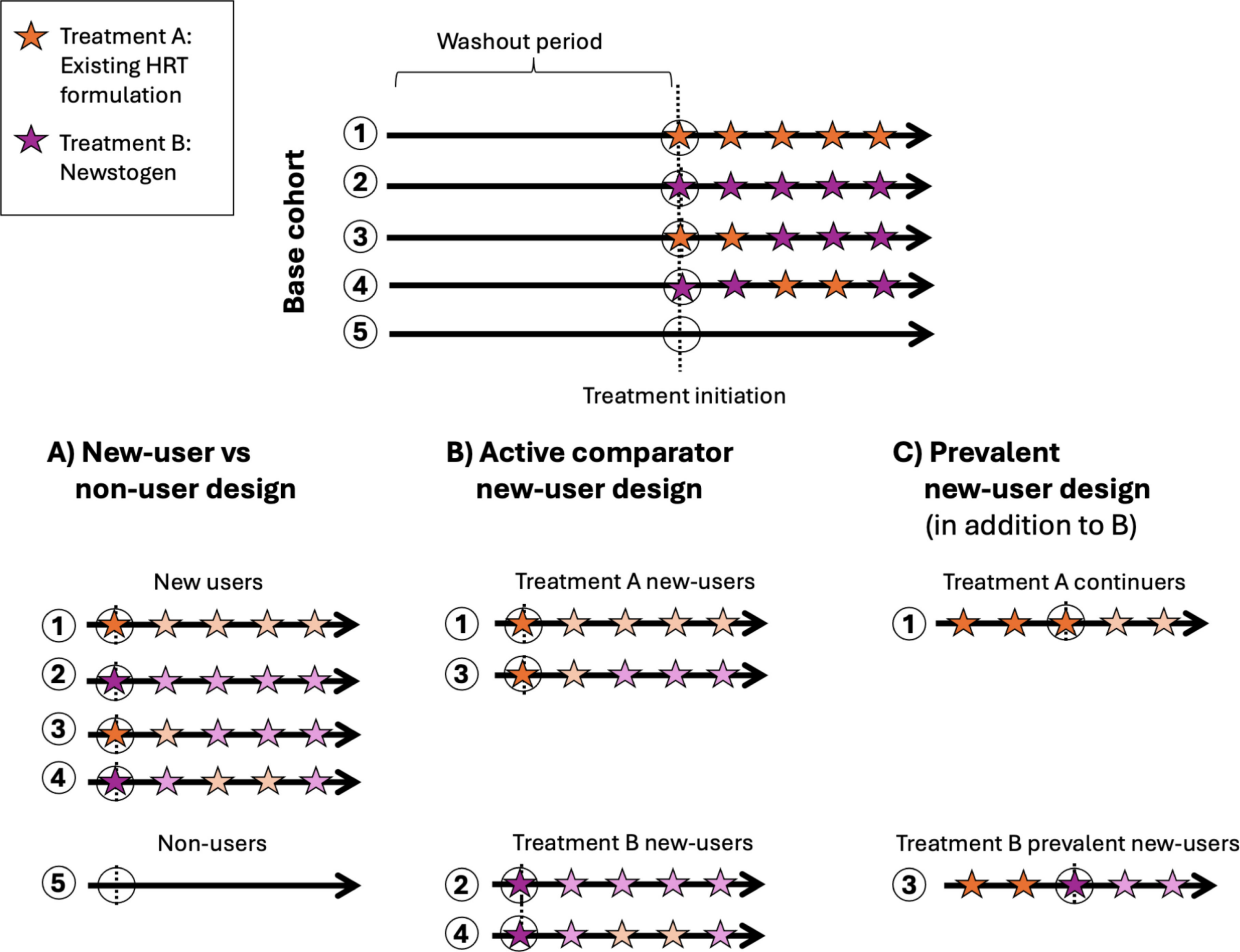
Graphical depiction of new‐user designs. This figure depicts the three new‐user study designs presented, showing which patient observations would be included from an illustrative underlying base cohort of 5 patients. (A) Defines a new‐user versus non‐user design, comparing new users of the existing HRT formulation or Newstogen versus non‐users. (B) Defines an active comparator new‐user (ACNU) design, comparing news users of the existing HRT formulation versus new users of Newstogen. (C) Highlights the additional observations added to ACNU necessary to define a prevalent new‐user design. The dashed black line represents treatment initiation (e.g., menopause onset). Black circles represent index dates when follow‐up is started. The washout period defines a period when individuals did not receive treatment. Orange and purple stars represent times an individual used the existing HRT formulation and Newstogen, respectively. Finally, treatment is defined by the first treatment prescribed/dispensed, and discontinuation or switching is ignored (denoted by the shading of stars beyond the index date).

Following the approval of Newstogen, we may wish to confirm the results of the placebo‐controlled RCT within real‐world populations, for example, to investigate whether the same benefits apply in clinical practice. Whilst we could imagine a target trial where real‐world patients are randomized to either “initiate HRT” or “initiate placebo,” the lack of placebo prescriptions in the real world makes it impossible to emulate such a trial in an observational context. Instead, we can shift our focus to a different clinical question: imagining an alternative, more pragmatic, trial in which patients eligible for Newstogen are randomized to either initiate Newstogen (new users) or no therapy whatsoever (non‐users). Analytically, this can be achieved using a type of new‐user design (also called an incident‐user or initiator design), where the comparator group is a group of non‐users, as in Figure 1 [1, 2, 3]. We refer to this as a new‐user versus non‐user design.

### 3.2 Design Overview

Common usage of the term “new‐user design” began following a 2003 paper by Wayne Ray (although the concepts underlying it are far older) [4, 15]. Given a population of interest, a new‐user design identifies patients initiating treatment (i.e., “new users”), and follow‐up begins from time of treatment initiation until observation of a relevant endpoint (e.g., health outcome, death, lost‐to‐follow‐up). New use is typically defined as having a minimum period of non‐use prior to initiation of treatment commonly referred to as the washout period (e.g., 12 months of no prescription claims for Newstogen or existing alternative HRT formulations). Researchers should also implement a minimum period prior to the start of follow‐up, equal to or longer than the washout period, in which to identify patient baseline characteristics (often referred to as a look‐back period or baseline covariate assessment window). As with other pharmacoepidemiology study designs, it is also important to consider that outcomes where detection bias could threaten validity may also require a lag period (e.g., where the person‐time at risk and counts of endpoints start 6‐months after initiation of Newstogen), and this requires careful consideration in the design and analysis [16]. Furthermore, lag periods (and the decisions made during them) can modify the causal question under study.

The new‐user versus non‐user design evaluates the following causal question: *what will be the difference in outcomes if patients initiate treatment compared with if they do not?* The fact that all patients begin follow‐up with an identical (or similar) recent treatment history at baseline helps avoid issues arising from the inclusion of prevalent users, including the depletion of susceptibles (i.e., the loss of patients at high risk for an outcome in the early portion of follow‐up) [17, 18], left‐truncation (i.e., time periods where exposure has begun but patients have yet to enter the study cohort) [1], and adjustment for intermediate variables (i.e., variables that are affected by initiating treatment that are on the causal path to the outcome) [19]. If non‐users are identified correctly, it also helps avoid immortal time bias (in which, by design, person‐time where outcomes cannot occur is incorrectly treated as time at risk) [20, 21]. Immortal time bias can also occur when person‐time is misclassified or excluded.

However, there are limitations. Perhaps the greatest limitation of new‐user versus non‐user designs is that comparisons to non‐users make it difficult to address confounding by disease severity (often referred to as *confounding by indication*) [6]. Also, restriction to new users often reduces sample size and the precision of estimated treatment effects compared with methods that attempt to incorporate person‐time from individuals with prior use of treatment [2]. This may be particularly important when individuals are frequently entering and leaving data sets (as in insurance claims data) or when studying a novel therapy, like Newstogen, where many people may be switching from an older HRT therapy.

Another key issue with the new‐user versus non‐user design surrounds defining the start of follow‐up for the non‐user comparator group. Several valid and simple approaches exist, including choosing the first time an untreated patient is eligible or matching or randomly sampling a subset of patients available at the treatment initiation times [8]. However, more general solutions (that allow the same patient to be considered for inclusion multiple times) include using sequential trial designs or clone‐censor‐weighting approaches, which can also accommodate more complex research questions, for example, surrounding treatment duration or initiation within a specified period (“initiate within 30‐days of diagnosis”), provided immortal time bias is avoided and the database includes sufficient information to model additional complexities, for example, surrounding time‐varying reasons for censoring [22, 23, 24, 25, 26].

## 4 Active Comparator New‐User Design

### 4.1 Motivation

Following the approval of Newstogen, we may also be interested in whether trial results have translated to real‐world reductions in cardiovascular events among “Newstogen initiators” compared with “alternative HRT initiators.” Since the large Phase III RCT of Newstogen was placebo‐controlled, its findings cannot directly address this type of question without making major assumptions about the external validity of the trial. This is particularly important given that we are comparing one treatment option with a cardiovascular benefit (Newstogen) to a treatment option with an adverse cardiovascular effect (existing alternative HRT formulations). One option to answer this question would be to emulate a target trial randomizing HRT‐naïve patients to either initiate Newstogen or an existing alternative HRT formulation with the same (or very similar) indication and disease severity as Newstogen. Analytically, this can best be achieved with an active comparator new‐user design (see Figure 1).

### 4.2 Design Overview

The active comparator new‐user design answers distinct causal questions while mitigating several of the issues with the new‐user versus non‐user design. The main addition to the new‐user versus non‐user design is the use of an active comparator rather than using non‐users as a comparator group. The active comparator is traditionally another treatment for the same (or very similar) indication used in similar patients (i.e., patients at “clinical equipoise”). Patients are required to meet washout criteria for **both** treatments of interest, that is, be naive to both therapeutic options during the washout period (this requirement may also extend to other treatments, for example, for the same indication, depending on the research question). Instead of examining the difference in outcomes if patients initiate treatment versus do not initiate treatment, the active comparator new‐user study design examines the differences in outcomes if patients initiate one treatment versus a therapeutic alternative.

The active comparator new‐user design maintains many of the advantages of new‐user designs more generally, but its single greatest additional advantage is its ability to mitigate confounding by indication when an appropriate active comparator is selected. For example, using an existing HRT formulation as the comparator would be appropriate if the formulation is prescribed for the same or (very similar) indication (e.g., women entering menopause at a similar age with similar symptom severity). This tends to mitigate confounding by measuring and unmeasured confounding variables that would threaten the validity of a new user study [27], though it is still important to include analytic strategies that adjust for any remaining differences between treatment groups. Indeed, it has been shown that well‐designed population‐based studies using an active comparator can enhance the validity and reduce the threat of confounding by indication [28]. Despite this, in practice, a novel treatment may not be fully interchangeable with an active comparator for the same indication, for example, if the novel treatment is preferentially prescribed to sicker or healthier patients, potentially leading to residual confounding. Finally, the use of active comparators usually avoids the issues new‐user versus non‐user study designs face with defining the appropriate start of follow‐up for non‐users and the resulting potential for the inclusion of immortality time bias. Despite this, immortal time may occur depending on how prior treatment to the comparator is captured; for example, see Suissa et al. discussion of “hierarchically” defined treatment groups [20, 29].

Active comparator new‐user designs do possess their own limitations. First, they require the existence of an actual active comparator. In cases where no such comparator exists (e.g., when attempting to compare statin use vs. non‐use) an “inactive active” comparator can be used if available [30]. Inactive comparators are medications that do not affect the risk of the outcome and whose indications do not affect the risk of the outcome. Whilst inactive comparators may attenuate some types of confounding (e.g., confounding due to frailty or healthcare utilization), they may not fully mitigate confounding by indication [30, 31]. Second, non‐use will often be more common than new use of any treatment, meaning the sample size limitation encountered by new user studies is only exacerbated in the context of an active comparator new user study, where new use is required in both treatment and comparator. Finally, like new‐user versus non‐user designs, active comparator new‐user designs focus on treatment initiation, with limited ability to study treatment switching or augmentation.

## 5 Prevalent New‐User Design

### 5.1 Motivation

Suppose we are interested in whether those currently taking HRT (who may greatly outnumber the number of new users of HRT) would benefit from switching to Newstogen. Given the complex relationship between time on HRT and cardiovascular risk [13], the treatment effects of switching may substantially differ from the treatment effects of initiation, meaning our active comparator new‐user study may not be a valid estimate of the potential effect of switching treatment. Additionally, we may expect a large proportion of potentially eligible people to be on an existing HRT formulation, and these people would be excluded from an active comparator new‐user study because they are non‐naive to treatment, likely impacting the generalizability of findings.

Therefore, instead of emulating a target trial randomizing patients to initiating Newstogen versus initiating existing alternative HRT formulations, we can instead emulate a target trial randomizing all individuals considering initiating Newstogen to start Newstogen or take an existing alternative HRT formulation (meaning Newstogen initiators with no history of HRT use are compared with new users of the existing HRT formulations, while those switching to Newstogen are compared to those who continued on their existing alternative HRT formulation); analytically, this can be achieved using the prevalent new‐user design [5].

### 5.2 Design Overview

The prevalent new‐user design was proposed to expand the population of inference in pharmacoepidemiology studies to include those who switched from standard of care treatments to novel interventions and increase the sample size of studies of drugs that recently entered the market [5, 32]. Creating a prevalent new user cohort is more complex than the cohorts described in the previous sections. The first two steps are identical to an active comparator new user study. First, specify the treatment (e.g., Newstogen) and a comparator of interest (e.g., older HRT). Second, identify incident new users of the two treatments with no history of the treatment or comparator (i.e., the cohort that would contribute to a typical active comparator/new‐user study). The incident new users of the comparator are used to estimate the counterfactual outcomes of the incident new users of the treatment of interest. Things now begin to diverge from an active comparator study as researchers must consider those who switch to Newstogen after initiating an older HRT.

This requires creating new observations when incident new users of the comparator (i.e., existing alternative HRT formulation) continue using the comparator or initiate the treatment of interest (i.e., Newstrogen), updating any time‐varying covariates, and re‐applying inclusion and exclusion criteria (meaning we would create new observations for each time someone refilled their older HRT or filled a new prescription for Newstogen instead of their old HRT). The observations where people initiate the treatment of interest become the target population and are referred to as “prevalent new users” to distinguish them from “incident new users” in the active comparator new‐user study design section of the study.

The final stage of cohort creation entails creating exposure sets that consist of prevalent new users and those with similar exposure histories who, instead of initiating the treatment of interest, continued use of the comparator to prevent exposure history from confounding our estimates (see Figure 1C for an example exposure set). These exposure sets may be based on calendar time, number of prescriptions, past adherence, or all three [33]; optimally, they should distinguish “direct switchers” from the comparator to the treatment of interest from “delayed switchers” who spend time off the comparator treatment from other more “complex switchers.” [28] In the case of Newstogen initiators, we would probably limit our prevalent new users of Newstogen to those who switched directly from older HRT to Newstogen. The exposure sets are the reason that prevalent new users can be included in the study without generating biases related to the depletion of susceptibles, left truncation, and adjustment for intermediate variables.

There are a variety of other major considerations and limitations of prevalent new‐user studies. First, the washout period and comparator choice are just as essential for prevalent new‐user studies as the aforementioned study designs. If a selected washout period is too short, there may be depletion of susceptibles. If an inappropriate comparator is selected, there may be confounding indication. Second, the selection of analytic methods (matching vs. weighting vs. outcome modeling) becomes more complex due to the need to incorporate the exposure sets [5]. If the exposure sets are not suitably implemented, left truncation bias and depletion of susceptibles can threaten the validity of the findings in the same way they threaten comparisons of exposed and unexposed person‐time. Third, differential surveillance patterns may exist between switchers and continuers. Therefore, investigators must consider the ability to capture key time‐varying information, for example, surrounding diagnosis of comorbidities, in both treatment groups. This concern can be mitigated by adding a healthcare‐contact component to the exposure set definition, for example, comparators are required to have a general practice visit within 3 months of the switcher prescription. Fourth, researchers must decide on whether it is clinically appropriate to combine the estimate from the incident new users with the estimate from the prevalent new users or whether such a combination ignores potential heterogeneity [34]. Finally, researchers need to carefully consider the appropriate time windows for matching or stratification, which types of switchers (direct vs. delayed vs. complex) represent the most appropriate study population, and what types of grace periods adequately capture the difference between these varying types. Different decisions reflect different assumptions about the association between exposure history, switching, and the outcome.

## 6 Conclusion

We reviewed three new‐user study designs commonly used in pharmacoepidemiology research and explored how each design emulates a distinct target trial within the target trial framework. This framework clarifies the research question, whether it involves comparing the initiation of one treatment versus a placebo (e.g., new‐user vs. non‐user), the comparison between initiating two distinct treatments (e.g., active comparator new‐user), or examining the effects of switching or discontinuing treatments (e.g., prevalent new‐user). Using this framework also necessitates articulation of the assumptions underlying our observational studies, such as how closely they can mimic an ideal randomized trial and the assumptions made during the emulation process. Table 1 summarizes the key features of the study designs presented.

While this review centered on fundamental study design elements, we have not attempted to present a comprehensive set of considerations necessary for applying these designs to a specific research question. Several key issues that would require more detailed consideration when implementing any of these designs in practice are now briefly discussed. First, depending on the research question and target population of interest, suitable eligibility criteria would need to be applied to ensure correct identification of the relevant patient population. Second, we limited our discussion to questions pertaining to static interventions and did not consider dynamic or time‐varying treatment strategies [35]. Despite this, it is important to highlight that the study designs presented can be extended to address these complexities as well as a wide variety of other pharmacoepidemiologic questions, including deprescribing [36]. Third, wider consideration would be required surrounding discontinuation and treatment adherence/persistence. As pharmacoepidemiologists, our interest often extends beyond focusing on the effect of starting a treatment strategy alone (i.e., intention‐to‐treat analyses) to understanding the effect of starting and following a treatment strategy (i.e., per‐protocol, on‐treatment, or as‐treated analyses) [37]. These questions necessitate approaches that require additional statistical considerations beyond the scope of this article [38].

Finally, it is critical to remember that selection of the most appropriate study design is dictated by the specific research question at hand. By focusing on a hypothetical target trial we would like to conduct and identifying the study design that emulates that trial, pharmacoepidemiologists can ensure they are answering relevant causal questions whilst also avoiding threats to validity.

## 7 Conflicts of Interest

The authors declare no conflicts of interest.
